# Electrolyzed Hydrogen Water Protects against Ethanol-Induced Cytotoxicity by Regulating Aldehyde Metabolism-Associated Enzymes in the Hepatic Cell Line HepG2

**DOI:** 10.3390/antiox10050801

**Published:** 2021-05-19

**Authors:** Satoshi Yano, Jinyun Wang, Shigeru Kabayama, Taichi Hara

**Affiliations:** 1 Laboratory of Food and Life Science, Faculty of Human Sciences, Waseda University, Tokorozawa 359-1192, Japan; s.yano3@kurenai.waseda.jp (S.Y.); wangjinyun@ruri.waseda.jp (J.W.); 2 Nihon Trim Co. Ltd., Oyodonaka, Kita-ku, Osaka 531-0076, Japan; kabayama@nihon-trim.co.jp

**Keywords:** electrolyzed hydrogen water, hydrogen, alcoholic liver disease (ALD), reactive oxygen species (ROS), alcohol dehydrogenase (ADH), aldehyde dehydrogenase (ALDH), alcohol toxicity

## Abstract

Excessive alcohol consumption can cause multi-systemic diseases. Among them, alcoholic liver disease is the most frequent and serious disease. Electrolytic hydrogen water (EHW) is produced at the cathode during electrolysis of water and contains a large amount of molecular hydrogen and a low content of platinum nanoparticles with alkaline properties. In this study, we found that EHW inhibits ethanol-induced cytotoxicity by decreasing the intracellular acetaldehyde, a toxic substance produced by ethanol degradation, in hepatocyte cell lines HepG2. Analysis of the mechanism of action revealed that EHW inhibits the metabolism of ethanol to acetaldehyde by suppressing alcohol dehydrogenase. EHW also promotes the metabolism of acetaldehyde to acetic acid by activating aldehyde dehydrogenase, which plays to reduce aldehyde toxicity and intracellular reactive oxygen species in HepG2 cells. These functions were correlated with the concentration of molecular hydrogen in EHW, and were abolished by degassing treatment, suggesting that molecular hydrogen may contribute as a functional factor in the suppression of ethanol-induced hepatocellular damage. Furthermore, hydrogen water with high dissolved hydrogen molecule showed the same hepatocellular protective effect against ethanol as the EHW. These results suggest that EHW may be useful in the prevention of alcoholic liver disease.

## 1. Introduction

Electrolytic hydrogen water (EHW) contains abundant molecular hydrogen and very small amounts of platinum nanoparticles with alkaline properties [1,2]. EHW and hydrogen water (water rich in molecular hydrogen) have been shown to have characteristics that scavenge reactive oxygen species (ROS) [2]. EHW has also been shown to reduce intracellular ROS levels in the fibrosarcoma cell line, HT1080, when exposed to H_2_O_2_ as an oxidative stress agent, with stronger activity than hydrogen water containing the same concentration of molecular hydrogen [2]. These EHW and hydrogen water properties have been verified by animal experiments to contribute to the alleviation of pathological conditions such as diabetes, arteriosclerosis, and neurodegenerative diseases, which are closely related to oxidative stress [3,4,5]. A recent study also showed that in a rat model of sustained stress-loading, pre-treatment with EHW significantly suppressed the elevation of peroxides, prevented the decline in antioxidant capacity in the blood, and significantly suppressed the elevation of inflammatory marker, IL-1β, and stress response hormone, ACTH [6]. In addition, a non-randomized, non-blinded, prospective observational study reported that hemodialysis using a dialysis solution containing high concentrations of hydrogen produced by electrolysis can reduce the mortality risk and cardiac and cerebrovascular complications in dialysis patients [7]. EHW also significantly reduces the energy expenditure in triathletes during endurance exercise [8].

The medical complications associated with alcohol consumption encompass a wide range of areas, among which is alcoholic liver disease (ALD). ALD includes alcoholic steatosis, alcoholic hepatitis, fibrosis, cirrhosis, and hepatocellular carcinoma [9] and it is a relatively common, life-threatening disease. The liver is the main organ of alcohol metabolism in the human body. In the liver, alcohol is metabolized into highly toxic acetaldehyde, which is then broken down into non-toxic acetic acid. Persistent drinking of alcohol causes acetaldehyde to damage liver cells, leading to serious diseases such as alcoholic cirrhosis. Of note, alcohol (ethanol: CH_3_CH_2_OH, ethyl alcohol) causes poisoning of the central nervous system, but alcohol itself is not hepatotoxic. The biological response to ethanol is regulated by the processes of acetaldehyde production by alcohol dehydrogenase (ADH), the drug-metabolizing enzymes cytochrome P450 (CYP) 2E1 and catalase, and its metabolism to acetic acid by aldehyde dehydrogenase (ALDH). The mechanism of alcohol-induced hepatocellular damage is thought to involve the redox shift associated with ethanol metabolism, toxicity of acetaldehyde, increased oxidative stress due to reactive oxygen species (ROS) production, as well as microcirculatory disturbances and activation of the inflammation system. In addition, acetaldehyde is a highly reactive and unstable substance that binds to proteins and DNA to form adducts, resulting in glutathione depletion, lipid peroxidation, and mitochondrial damage [10]. 

In the past, there have been many studies on ALD. To date, there is no FDA-approved nutritional therapy for ALD, and liver transplantation is the ultimate treatment option for patients with advanced alcoholic cirrhosis [11]. Pre-drinking hydrogen water containing high concentrations of hydrogen has been reported to reduce the development of fatty liver (early lesions of ALD) in mice and the production of pro-inflammatory cytokines (such as IL6 and TNFα) in response to alcohol administration [12]. It has also been shown that the addition of hydrogen to acetaldehyde solution decreases the aldehyde concentration [13]. These results suggest that hydrogen has a detoxifying effect on acetaldehyde and may contribute to alleviating the pathology of alcoholic diseases. However, the mechanisms are largely unknown.

Although the antioxidant properties of EHW are well known, the effects of EHW on ethanol-induced hepatic cell damages have not been evaluated so far. In this study, we found that EHW suppressed cell death and ROS accumulation in the ethanol-treated hepatocyte cell line, HepG2, by decreasing intracellular acetaldehyde, a toxic substance produced by ethanol degradation. Analysis of the mechanism of action revealed that EHW inhibits the metabolism of ethanol to acetaldehyde by suppressing alcohol dehydrogenase. EHW also promotes the metabolism of acetaldehyde to acetic acid by activating aldehyde dehydrogenase (ALDH), which reduces intracellular toxic aldehyde levels in HepG2 cells. These functions were correlated with the concentration of molecular hydrogen in EHW, and were abolished by degassing treatment, suggesting that molecular hydrogen may contribute as a functional factor in the suppression of ethanol-induced hepatocellular damage. 

## 2. Materials and Methods

### 2.1. Different Types of Water 

EHW was obtained from a previously reported apparatus, TRIM ION GRACE (Nihon Trim Co., Osaka, Japan) [6]. In brief, tap water is first passed through an activated charcoal filter for removal of bacteria and other microscopic impurities, then subject to electrolysis for enrichment in hydrogen. The apparatus was used to generate 4 kinds of EHW (LV1-4) by varying the electric current. Filtered water (FW) that was not subjected electrolysis was used as a control. Additionally, EHW was subjected to different treatments to obtain EHW with specific physical and chemical compositions; EHW with neutral pH, high concentration of dissolved hydrogen, and a small amount of dissolved oxygen were prepared [3,14,15]. EHW (pH) was neutralized with HEPES buffer to adjust the pH. EHW (LV4) was autoclaved twice to generate autoclave water (AC). Hydrogen water (HW) with high dissolved hydrogen was obtained using a kit from TRIM SEVEN WATER (Nihon Trim). HW was prepared at the same hydrogen concentration as the EHW LV4. The concentration of dissolved hydrogen in fresh EHW was measured using a flow cell type hydrogen sensor (DH-35A, TOADKK, Tokyo, Japan). The dissolved hydrogen concentration of this study is as follows; LV1: 780–830 ppb, LV2: 850–880 ppb, LV3: 1060–1140 ppb, LV4: 1260–1350 ppb, and HW: 1300 ppb. The pH of FW and EHW (LV1-4) was measured using LAQUAact D-71 pH meter (HORIBA Advanced Techno, Co., Ltd., Kyoto, Japan), value of which is as follow; FW: 8.05 ± 0.02, LV1: 8.94 ± 0.04, LV2: 9.20 ± 0.04, LV3: 9.57 ± 0.12, LV4: 10.09 ± 0.02. 

### 2.2. Cell Culture

HepG2 cells (RIKEN BRC), a human liver cancer cell line, were cultured in Dulbecco’s Modified Eagle Medium (DMEM) (Wako, 044–29765) containing 10% fetal bovine serum (FBS; Sigma-Aldrich, F7524) and 1% penicillin-streptomycin (Wako, 168–23191) at 37 °C in a 5% CO_2_ atmosphere. Experimental 5× DMEM medium was prepared by D-MEM powder (Wako, 049-33561) and ultrapure water from a Milli-Q synthesis system (Millipore, Tokyo, Japan), which was diluted with FW, EHW, EHW (pH), AC, or HW, to prepare the treatment medium (20%, 5× DMEM, 80%, FW, EHW, EHW (pH), AC, or HW). 

### 2.3. Measurement of the Inhibitory Effect on Cell Proliferation

HepG2 cells were seeded in a 24-well plate at a density of 5.0 × 10^4^ cells in DMEM and cultured for 24 h in the CO_2_ incubator. The cells were treated with medium containing FW, EHW, EHW (pH), AC, or HW spiked with ethanol or acetaldehyde. The cells were incubated for 24 h. Then, the cells were stained with trypan blue (Wako, 207–17081), and unstained cells were counted as alive under an inverted microscope, and the cell survival rate was calculated.

### 2.4. Measurement of Intracellular ROS 

HepG2 cells were seeded in a 12-well plate at a density of 1.0 × 10^5^ cells/well for 24 h, treated with ethanol or acetaldehyde in a medium prepared with FW, EHW, EHW (pH), AC or HW for 6 h. Then, the cells were treated with 5 μM intracellular ROS fluorescence detection reagent CM-H_2_DCFDA (Thermo Fisher Scientific, C6827) for 30 min. The fluorescence in the cells was visualized by fluorescence microscope (KEYENCE BZ-9000), and the fluorescence intensity was measured using Cellometer^®^Vision (Nexcelom Bioscience LLC) after harvest. FCS Express4 (De Novo software) was used for quantitative analysis.

### 2.5. Measurement of the Concentration of Ethanol and Acetaldehyde

Ethanol or acetaldehyde were added to FW, EHW (LV4), AC, or HW to prepare 4% ethanol and 1 mM acetaldehyde solutions in 50 mL tube, respectively. The solutions were stored at room temperature for 24 h. Then, the ethanol concentration was measured using the QuantiChrom™ Ethanol Assay Kit (BioAssay Systems, DIET-500), according to the manufacturer’s protocol. Briefly, 100 µL of these waters that were mixed with 4% ethanol and standard were added to each well at 96-well plate, followed by addition of 100 µL of Reagent A, colorimetric assay buffer. After incubation at room temperature for 8 min, the absorbance was determined by measuring at 570 nm with Multiskan™ FC (Thermo Scientific™, MA, USA). Additionally, the acetaldehyde concentration was measured using the EnzyChrom™ Acetaldehyde Assay Kit (BioAssay Systems, EACT-100) according to the manufacturer’s protocol. Briefly, 20 µL of these waters that were mixed with 1 mM acetaldehyde and standard were added to each well at 96-well plate, followed by addition of 80 µL of Warking Reagent, including Assay buffer, NAD/MTT solution, Enzyme A, and Enzyme B. After incubation at room temperature for 30 min, the absorbance was determined by measuring at 570 nm with Multiskan™ FC.

### 2.6. Measurement of the Ethanol Concentration in Culture Medium

HepG2 cells were seeded in a 6-well plate at a density of 2.0 × 10^5^ cells/well for 24 h. Then, the cells were treated for 24 h with medium that was mixed with 4% ethanol and FW, EHW (LV4), AC, or HW. Ethanol concentration in the culture medium was measured using the QuantiChrom™ Ethanol Assay Kit (BioAssay Systems, DIET-500) according to the manufacturer’s instruction. Briefly, the culture medium was deproteinated by adding 1 volume of these medium to 2 volumes of 10% TCA, followed by centrifuge at 14,000 rpm for 5 min, and their supernatant was used for the assay. Colorimetric quantification was performed in the same way as in Section 2.5.

### 2.7. Measurement of Acetaldehyde Concentration in Cells

HepG2 cells were seeded in a 10 cm dish at a density of 1.0 × 10^6^ cells/well for 24 h. Cells were incubated for 24 h with medium that was mixed with 1 mM acetaldehyde and FW, EHW (LV4), AC, or HW. The intracellular acetaldehyde concentration was measured using the EnzyChrom™ Acetaldehyde Assay Kit (BioAssay Systems, EACT-100) according to the manufacturer’s instruction. Briefly, the cells were lysed and sonicated, followed by centrifuge at 12,000 rpm, for 5 min, and their supernatant was used for the assay. Colorimetric quantification was performed in the same way as in Section 2.5.

### 2.8. Measurement of Cellular ADH Activity 

HepG2 cells were seeded in a 6-well plate at a density of 2.0 × 10^5^ cells/well for 24 h. Then, the cells were incubated for 24 h with medium that was mixed with 4% ethanol and FW, EHW (LV4), AC, or HW. The cellular ADH activity was measured using the Alcohol Dehydrogenase Activity Colorimetric Assay Kit (BioVision, K787-100) according to the manufacturer’s instruction. Briefly, the cells were lysed with ADH Assay Buffer, 50 µL of which were mixed with 100 µL of Reaction Mix, including ADH Assay Buffer, Developer, and Substrate, to each well at 96-well plate. The mix was incubated for 3 min at 37 °C, then the absorbance was measured at 450 nm with Multiskan™ FC, which was expressed as Abs_0min_, and was incubated for another 60 min at 37 °C and the absorbance was measured at 450 nm again, which was expressed as Abs_60min_. The ∆Abs (= Abs_60min_ − Abs_0min_) was applied to the NADH standard curve to get X nmol of NADH generated by ADH during the reaction time (60 min). The ADH activity was calculated from the following equation:

ADH activity = X nmol/ (60 min × 0.15 mL) (nmol/min/mL = mU/mL)

Then, the cell lysate volume was converted to the number of cells and counted by trypan blue method, and the ADH activity was recalculated (µU/1.0 × 10^6^ cells).

### 2.9. Measurement of Cellular ALDH Activity

HepG2 cells were seeded in a 6-well plate at a density of 2.0 × 10^5^ cells/well for 24 h. Then, the cells were incubated for 24 h with medium that was mixed with 4% ethanol and FW, EHW (LV4), AC, or HW. The cellular ALDH activity was measured using the Aldehyde Dehydrogenase Activity Colorimetric Assay Kit (BioVision, K731-100) according to the manufacturer’s instruction. Briefly, the cells were lysed with ALDH Assay Buffer, 50 µL of which were mixed with 50 µL of ALDH Measurement, including ALDH Assay Buffer, ALDH Substrate Mix, and Acetaldehyde, to each well at 96-well plate. The mix was incubated for 5 min at room temperature, then the absorbance was measured at 450 nm with Multiskan™ FC, which was expressed as Abs_0min_, and was incubated for another 60 min at room temperature and the absorbance was measured at 450 nm again, which was expressed as Abs_60min_. The ∆Abs (= Abs_60min_ − Abs_0min_) was applied to the NADH standard curve to get X nmol of NADH generated during the reaction time (60 min). The ALDH activity was calculated from the following equation:

ALDH activity = X nmol/ (60 min × 0.10 mL) (nmol/min/mL = mU/mL)

Then, the cell lysate volume was converted to the number of cells and counted by trypan blue method, and the ALDH activity was recalculated (µU/1.0 × 10^6^ cells).

### 2.10. Statistical Analysis

The data represent the mean ± SEM. Statistical analysis was performed by two-tailed Student’s *t*-test, or one-way analysis of variance (ANOVA), and then, Dunnett’s test and Tukey’s test were used as a multiple comparison test. A probability of *p* < 0.05 was considered as significant. 

## 3. Results

### 3.1. Ethanol Induces Cell Death

**Figure 1:**
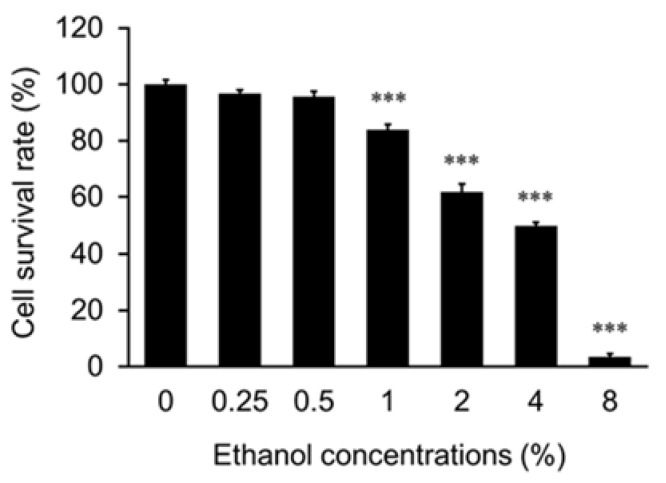
Measurement of cell viability of HepG2 following ethanol treatment. The cell survival rate was measured by the trypan blue method following treatment of HepG2 cells with different concentrations of ethanol. The data represent the mean ± SEM (*n* = 3). Asterisks indicate significant difference analyzed with Dunnett’s test. *** *p* < 0.001 vs. 0% ethanol as a control.

To evaluate whether EHW has a protective effect on alcohol-induced cell death, we first determined the optimal concentration of ethanol in the hepatocellular carcinoma cell line, HepG2, which is commonly used in toxicity studies [16]. As shown in Figure 1, the cell survival rate gradually decreased as the ethanol concentration increased in HepG2 cells treated for 24 h. Thus, ethanol was found to decrease the cell survival in a concentration-dependent manner. At an ethanol concentration of 4%, the cell survival rate was half that of 0% ethanol treatment as a control group. For further studies to evaluate the hepatoprotective effects of EHW, 4% ethanol was used.

### 3.2. EHW Protects HepG2 Cells from Ethanol-Induced Cell Death

**Figure 2:**
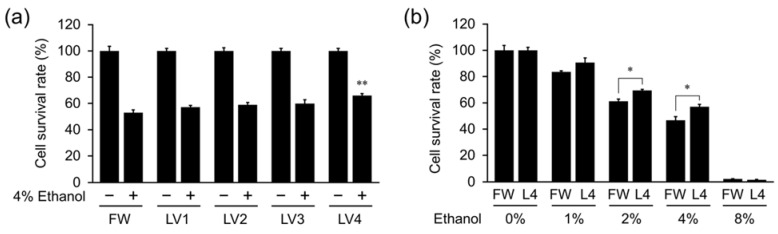
Measurement of cell viability of HepG2 following ethanol—EHW co-treatment. The cell survival rate of HepG2 cells were measured by the trypan blue method after culture in a medium prepared using filtered water (FW) or EHW of different grades (LV1–4), prepared using different electric currents (0.8–4.2 A) and then subjected to 4% ethanol (**a**), and FW or EHW LV4 containing different concentrations ethanol (**b**). The cells were incubated for 24 h following ethanol treatment. The data represent the mean ± SEM (*n* = 3). Asterisks indicate significant difference analyzed with Dunnett’s test, ** *p* < 0.01 vs. FW as a control among the 4% ethanol treatment groups (**a**), or Student’s *t*-test, * *p* < 0.05 vs. FW at different concentrations ethanol (**b**).

To investigate the protective effect of EHW on HepG2 cells from ethanol-induced cell death, cells were treated with 4% ethanol mixed with EHW of different grades (LV1-4) or filtered water (FW, without electrolysis) as the control for 24 h, and then the cell survival rate was calculated. As shown in Figure 2a, the cell survival rate which is calculated by converting each group without 4% ethanol treatment as 100% control, indicated that a slight increase with the intensity of electric current used for preparing EHW in 4% ethanol mixed, and there was a significant difference between EHW LV4 and FW in ethanol-treated cells. Thus, this cellular protective ability is closely correlated with electrolysis levels.

To better explore the relationship between EHW and ethanol-treated cells, we used ethanol concentrations from 1 to 8%. As shown in Figure 2b, the cell survival rate revealed that at 2% and 4%, EHW LV4 (L4) cells had a higher cell survival rate than FW cells, at the same ethanol concentrations. The difference was the most evident when the ethanol concentration was 4%. The results indicated EHW LV4 and 4% ethanol were the most appropriate condition options to be used in the following experiments.

### 3.3. Dissolved Gases, and Not Alkalinity, Is Responsible for EHW Protective Effects

**Figure 3:**
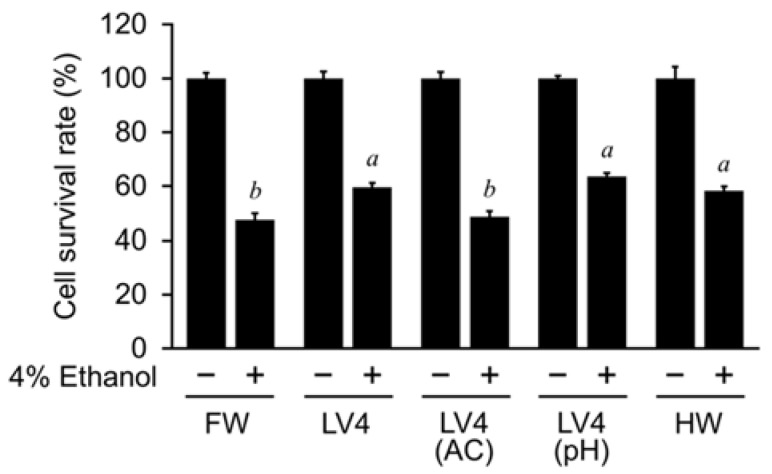
Evaluation of hepatoprotective effects of treated different water samples. The cell survival rate of HepG2 cells determined by the trypan blue method after cultured in medium containing filtered water (FW), untreated EHW LV4 (LV4), autoclaved EHW LV4 [LV4 (AC)], pH adjusted neutral EHW LV4 [LV4 (pH)], or hydrogen rich water (HW), with or without 4% ethanol. The data represent the mean ± SEM (*n* = 3). Data were analyzed by Tukey’s test after one-way ANOVA (*p* < 0.05). Different letters above the bars indicate significant difference among treatments for each water with 4% ethanol.

A recent study found that EHW is alkaline and has high concentrations of dissolved hydrogen [17]. To determine which of these two major characteristics of EHW is responsible for its protective ability, we treated EHW LV4 to obtain autoclaved EHW [LV4 (AC)] or neutralized EHW [LV4 (pH)]. The autoclaving steps are thought to remove components that are not heat-resistant and not highly pressure-resistant in EHW. FW and untreated fresh EHW LV4 were used as controls. Additionally, we prepared hydrogen rich water (HW) at the same level of hydrogen dissolved to EHW LV4. As shown in Figure 3, the cell survival rate was increased in cells treated with EHW LV4 compared with FW, in a 4% ethanol mixed condition. On the other hand, this effect was unobservable in cells treated with LV4 (AC), which were decreased to the same level as the control group. In contrast, the effect of EHW LV4 was still observed in cells treated with LV4 (pH). Moreover, HW also appeared to increase the number of viable cells and the cell survival rate in the 4% ethanol mixed condition. The results suggest that the large amount of hydrogen dissolved in water may be related to the protective effect of EHW.

### 3.4. EHW Suppresses Elevated ROS Levels in HepG2 Cells Treated with Ethanol 

**Figure 4:**
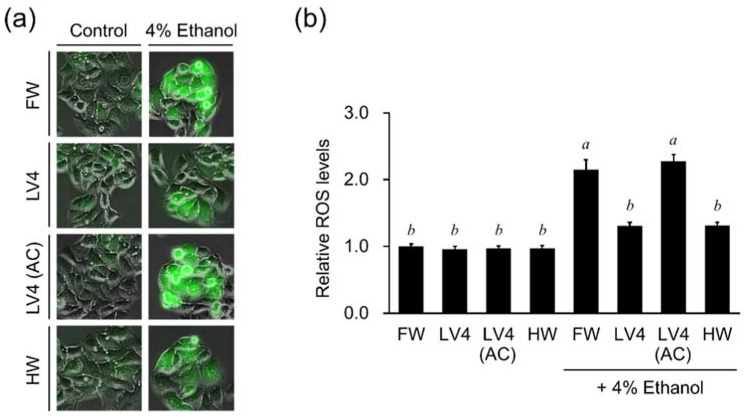
The effect of different water samples on the intracellular ROS levels after ethanol treatment in HepG2 cells. (**a**) Fluorescence microscopy images. (**b**) Relative ROS levels in cells. HepG2 cells were treated with 4% ethanol in medium containing filtered water (FW), untreated EHW LV4 (LV4), autoclaved EHW LV4 [LV4 (AC)], or hydrogen rich water (HW) for 6 h, and then stained with 5 μM of CM-H_2_DCFDA for 30 min. The data represent the mean ± SEM (*n* = 3). Data were analyzed by Tukey’s test after one-way ANOVA (*p* < 0.05). Different letters above the bars indicate significant difference among treatments for each water with or without 4% ethanol.

EHW has been shown to exert superior ROS-scavenging activities in HT1080 cells [2]. To further evaluate the relationship between EHW protective ability and its ability of decreasing ROS levels in hepatic cell culture models, we measured the cellular ROS levels in HepG2 cells. As shown in Figure 4a, the fluorescent signal of ROS in cells treated with FW and autoclaved EHW [LV4 (AC)] were substantially higher than that in EHW LV4 and HW under the 4% ethanol condition. As indicated in Figure 4b, no differences were observed between relative ROS levels in FW, EHW LV4, LV4 (AC), and HW-treated cells. In contrast, 4% ethanol increased relative ROS levels in all groups, particularly in the FW and LV4 (AC) groups. Moreover, EHW LV4 and HW cells that had been exposed to ethanol had significantly lower levels of ROS compared to the control (Figure 4b). Overall, the results indicated that the effect of EHW on the reduction of ROS levels in cells is related to the large amount of hydrogen dissolved in the water. 

### 3.5. EHW Protects HepG2 Cells from Acetaldehyde-Induced Intracellular ROS Elevation and Cell Death

**Figure 5:**
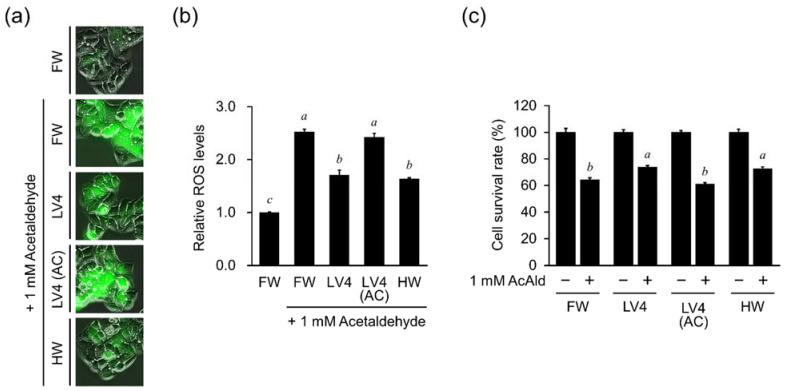
Protective effect of EHW on HepG2 cells treated with acetaldehyde. (**a**) Fluorescence microscopy images. (**b**) Relative ROS levels in cells. Cells were stained with 5 μM of CM-H_2_DCFDA for 30 min after cell culture in the presence of filtered water (FW), untreated EHW LV4 (LV4) and autoclaved EHW [LV4 (AC)] and hydrogen rich water (HW) with or without 1 mM acetaldehyde. (**c**) The cell survival rate was measured by the trypan blue method. The data represent the mean ± SEM (*n* = 3). Data were analyzed by Tukey’s test after one-way ANOVA (*p* < 0.05). Different letters above the bars indicate significant difference among treatments for each water with or without 1 mM acetaldehyde treatment (**b**) or among treatments for each water with 1 mM acetaldehyde treatment (**c**).

We next analyzed whether EHW and hydrogen water exert cytoprotective effects against aldehydes, the main toxic metabolite of ethanol toxicity. First, we detected the ROS levels in cells treated with acetaldehyde. As shown in Figure 5a, when cells were treated with 1 mM acetaldehyde, ROS fluorescent signals in medium containing EHW LV4 and HW were substantially lower in that in FW and autoclaved EHW [LV4 (AC)]. Furthermore, the quantitative analysis in Figure 5b indicated that the increase of ROS levels by 1 mM acetaldehyde was reduced significantly in medium containing EHW LV4 and HW, but not LV4 (AC). The results suggest that the effect of EHW on the reduction of ROS levels in cells is related to the large amount of hydrogen dissolved in water. Next, we evaluated the cytoprotective effect of EHW upon exposure to acetaldehyde. As shown in Figure 5c, the survival rate was significantly increased in medium with EHW LV4 and HW, but not LV4 (AC), compared with FW as a control, in the presence of 1 mM acetaldehyde. These results indicated that EHW has hepatocyte protective potency with reduction of intracellular ROS against acetaldehyde toxicity.

### 3.6. EHW Decreases Intracellular Acetaldehyde Levels in HepG2 Cells Treated with Ethanol 

**Figure 6:**
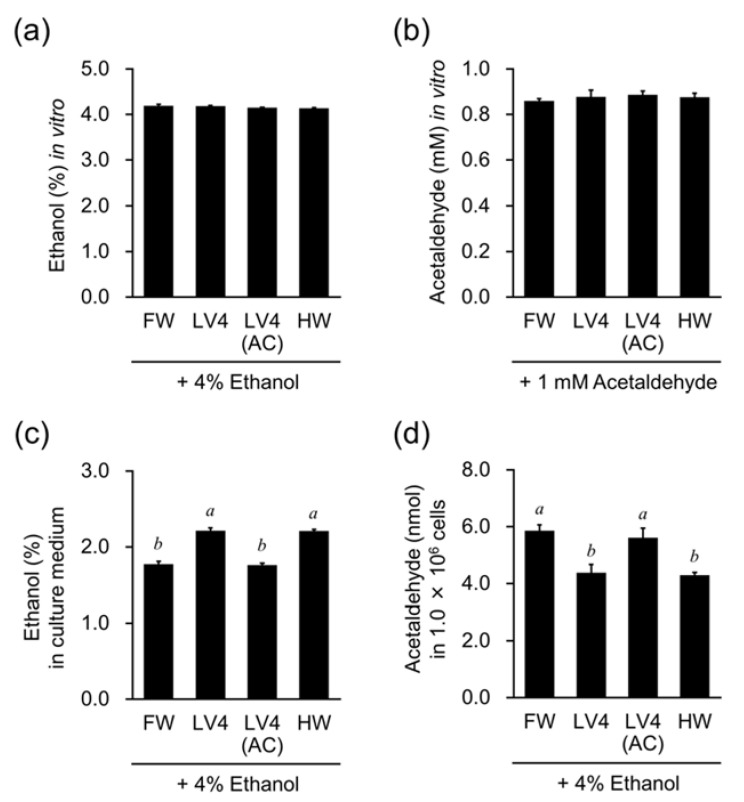
The comparison of the effect of EHW on ethanol and acetaldehyde metabolism in HepG2 cells. Concentration of ethanol (**a**) and acetaldehyde (**b**) in filtered water (FW), untreated EHW LV4 (LV4), autoclaved EHW [LV4 (AC)], or hydrogen rich water (HW) after 24 h. Ethanol concentration in cell culture supernatant (**c**), and concentration of acetaldehyde in cells (**d**) treated with FW, LV4, LV4 (AC), or HW mixed with 4% ethanol. The data represent the mean ± SEM (*n* = 3). Data are analyzed by Tukey’s test after one-way ANOVA (*p* < 0.05). Different letters above the bars indicate significant difference among treatments for each water with 4% ethanol.

To ascertain whether EHW exerts its effects via ethanol metabolism in HepG2 cells, the results were compared with that following ethanol treatment. First, different types of water with 4% ethanol or 1 mM acetaldehyde were prepared in tubes and stored at room temperature for 24 h. As shown in Figure 6a,b, the concentrations of ethanol and acetaldehyde did not show a significant difference in FW, EHW LV4, autoclaved EHW [LV4 (AC)], and HW after 24 h. Next, to examine the ethanol metabolism in HepG2 cells, the ethanol concentration in medium and the intracellular acetaldehyde concentration were measured. As shown in Figure 6c, the concentration of ethanol in medium was found to be increase in EHW LV4 and HW, but not LV4 (AC), compared to FW as the control. At the same time, the concentration of cellular acetaldehyde was found to be decreased in EHW LV4 and HW, but not LV4 (AC) (Figure 6d). Thus, these results suggest that EHW can inhibit the ethanol metabolism in HepG2 cells.

### 3.7. EHW Supresses ADH and Enhances ALDH Activities in the Ethanol Metabolism of HepG2 Cells

**Figure 7:**
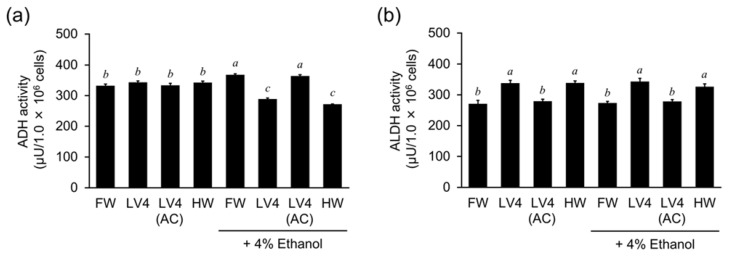
Effect of EHW on ADH activity and ALDH activity. (**a**) ADH activity and (**b**) ALDH activity in HepG2 cells cultured in the presence of filtered water (FW), untreated EHW LV4 (LV4) and autoclaved EHW [LV4 (AC)] and hydrogen rich water (HW) with and without ethanol treatment. The data represent the mean ± SEM (*n* = 3). Data were analyzed by Tukey’s test after one-way ANOVA (*p* < 0.05). Different letters above the bars indicate significant difference among treatments for each water with or without 4% ethanol.

Next, to elucidate the effect of hydrogen-dissolved water on ethanol metabolism in HepG2 cells, we measured the activity of alcohol dehydrogenase (ADH) and aldehyde dehydrogenase (ALDH). There are 19 isoforms of human ALDH. In particular, ALDH2 is known to act as the responsible enzyme in alcohol detoxification. ALDH1A1, ALDH1B1, ALDH3A1, ALDH3A2, ALDH3B1, and ALDH3B2 are also known to function as alcohol metabolizing ALDHs [18]. On the other hand, ALDH1A2 does not use acetaldehyde as its main substrate [18]. In this study, we evaluated the whole ALDH activity involved in the degradation of acetaldehyde, not only ALDH2. As shown as Figure 7a, ADH activity was increased in cells treated with 4% ethanol in medium containing FW and was decreased in medium containing EHW LV4 and HW, but not LV4 (AC). However, its activity was not affected by the presence of 4% ethanol. On the other hand, ALDH activity was increased in medium containing EHW LV4 and HW, but not LV4 (AC), compared with FW as a control. Moreover, the presence of 4% ethanol made no difference in these results. Thus, EHW inhibited the metabolism of ethanol to acetaldehyde by suppressing ADH activity and promoted the metabolism of acetaldehyde to acetic acid by increasing ALDH activity, suggesting a role in reducing aldehyde toxicity in HepG2 cells.

## 4. Discussion

In this study, we analyzed the protective effect of EHW against ethanol-induced hepatocellular damage and its mechanism using the hepatocyte cell line HepG2. We found that EHW can inhibit the increase in intracellular ROS levels caused by ethanol and aldehyde treatment, and prevent cell death, which is related to the electrolytic intensity of EHW. We also revealed that EHW decreases the highly toxic acetaldehyde in ethanol treated cells by inhibiting the activity of ADH, which is responsible for the production of acetaldehyde, and increasing the activity of ALDH, which is responsible for the degradation of acetaldehyde.

Unlike tap water, EHW has alkaline properties, including high concentrations of molecular hydrogen and trace amounts of platinum nanoparticles generated at the cathode by electrolysis of water. Previous reports have shown that EHW significantly inhibits the production of ROS in H_2_O_2_-treated HT1080 cells. This activity was found to be five times stronger than that of bubbled hydrogen water containing the same concentration of molecular hydrogen. In addition, about 60% of the activity of EHW remained even after degassing to remove hydrogen gas, speculating that a small amount of platinum nanocolloid in the electrolytic hydrogen water may contribute to the intracellular ROS scavenging ability by H_2_O_2_ treatment [2]. Similarly, the production of ROS is increased in HepG2 cells treated with ethanol and aldehyde, which inhibited by treatment of the cells with EHW or hydrogen water (Figure 4 and Figure 5). However, the cytoprotective and ROS scavenging effects of EHW against ethanol and aldehyde were abolished by degassing treatment but not by neutralization (Figure 3, Figure 4 and Figure 5). The same activity was observed in hydrogen rich water (Figure 3, Figure 4 and Figure 5), suggesting that the cytoprotective ability is related to the large amount of dissolved hydrogen molecules in the EHW. Whereas, since EHW and hydrogen water are also characterized by low dissolved oxygen [2], we cannot deny the possibility that low dissolved oxygen may have an effect on the increase in intracellular ROS and cytoprotective effects of ethanol and aldehydes. The production of ROS by ethanol and aldehydes occurs not only as a result of mitochondrial damage, but also as a major product or byproduct of various enzymatic reactions associated with alcohol degradation. In addition, it is known to induce disruption of the antioxidant system [19], suggesting that H_2_ molecules suppress ROS generation by acting on the intracellular ROS production mechanism caused by ethanol metabolism and aldehyde toxicity. On the other hand, since H_2_O_2_ is a precursor of hydroxyl radicals, components other than molecular hydrogen in EHW may act more strongly to scavenge intracellular reactive oxygen species caused by hydroxyl radicals [20]. Alternatively, the fibrosarcoma cell line, HT1080, and the hepatocyte cell line, HepG2, may differ in their responses to components in EHW in the production and scavenging mechanism of ROS, depending on the cell type. Further elucidation of the mechanism of action of EHW on intracellular ROS scavenging activity will be necessary to clarify these differences.

Oxidative stress has been shown to be deeply involved in the development of chronic liver diseases, including ALD [21]. The process of alcohol metabolism in cells causes the generation of ROS and also impairs the antioxidant system, resulting in oxidative stress that induces cellular damage [22]. EHW and hydrogen water markedly inhibit alcohol- and aldehyde-induced increases in ROS, suggesting that suppression of ROS production by molecular hydrogen may function as one of the mechanisms of action for cytoprotection (Figure 4 and Figure 5). However, aldehydes can react with various macromolecules such as lipids, proteins, and nucleic acids, impairing their functions and inducing strong cytotoxicity [23]. Thus, they may be considered a major factor in alcohol toxicity, affecting not only in the production and scavenging of ROS, but also in cellular homeostasis, producing a wide range of cytotoxic effects. In the present study, EHW and hydrogen water suppressed the production of intracellular aldehyde (Figure 6d). We suspect that the decrease in intracellular aldehydes may play a greater role in the cytoprotective effect than the inhibition of ROS production. Microcluster, a hydrogen-absorbing microporous silica, has been reported to reduce acetaldehyde in distilled spirits [13]. On the other hand, our in vitro analysis showed that the addition of ethanol or acetaldehyde to EHW or hydrogen water did not make any difference in their concentrations in the 24-h reaction (Figure 6a,b). There was no significant difference in the concentration of aldehydes used compared to previous reports (30 or 50 ppm vs. 44 ppm in this experiment). Since the microcluster continuously generates hydrogen gas in the aldehyde solution, the aldehyde concentration may be reduced by the continuous reaction of fresh hydrogen with the aldehyde. In fact, EHW or hydrogen water suppressed activity of ADH, the enzyme related to ethanol metabolism in ethanol-treated cells (Figure 7a), and increased the activity of ALDH, the enzyme responsible for the degradation of aldehydes (Figure 7b), suggesting that the regulation of alcohol metabolism plays an important role in reducing the amount of intracellular aldehyde. Therefore, although EHW and hydrogen water inhibit ROS production and cell death in aldehyde-treated cells (Figure 5), the degradation of aldehydes via activation of ALDH may contribute to these protective effects as a more fundamental mechanism of protection.

It is an important issue whether the inhibitory effect of molecular hydrogen on alcohol- and aldehyde-induced hepatocyte cytotoxicity, which was revealed in this study, can be observed in vivo. However, it has been reported that the development of fatty liver, which is an initial of alcoholic liver disease, and inflammatory reactions are suppressed in mice pretreated with hydrogen water [12]. In the future, it will be important to examine the effects of hydrogen water on the activity of ADH and ALDH in the liver in this model. Interestingly, although ADH and ALDH are different enzymes involved in the production and degradation of aldehydes, EHW acts positively and negatively on their respective activities to reduce the amount of aldehydes. Notably, electrolytic hydrogen water and hydrogen water reduced ADH activity only in the ethanol-treated HepG2 cells (Figure 7a), whereas ALDH activity was activated with or without ethanol treatment (Figure 7b), suggesting that molecular hydrogen may regulate the activity of each enzyme through indirect and/or direct mechanisms. However, the mechanism has not been clarified in this study. It has been reported that not only the activity of ADH but also mRNA levels and protein levels are altered in ethanol feeding experiments in rats [24]. In addition, an analysis using *Aldh2*-/- mice, in which human ALDH2 was overexpressed, reported that ethanol administration decreased the amount of acetaldehyde in the blood [25]. Whether EHW and hydrogen water directly regulate ADH and ALDH enzymatic activities, as well as gene expression and/or protein levels of ADH and ALDH, will need to be clarified in the future.

Moderate drinking of alcohol has been reported to be beneficial in reducing the risk of cardiovascular and all-cause mortality, which is widely accepted as a J-shaped curve association [26]. However, recent reports indicate that the increased risk of other diseases, including cancer, has been reported even for low levels of alcohol intake, indicating that there are no safe levels of alcohol consumption for improving health [27]. Thus, a two-sided effect of alcohol consumption on health has been reported, and this difference may be due to the balance between effects such as stress relief and platelet inhibition [28,29] by ethanol and cytotoxicity by aldehydes. The ability of EHW and hydrogen water to alter the balance between alcohol and toxic aldehyde levels by regulating ethanol metabolism may be a new preventive strategy to address health problems caused by alcohol consumption. In addition, it has been suggested that ALDH is involved in the metabolism of environmental toxins other than aldehydes [10], and if EHW and hydrogen water can increase ALDH activity in the body, it may also be effective in mitigating the toxicity of environmental factors other than alcohol.

## 5. Conclusions

Accumulating evidence has revealed that EHW have positive effects on health, but the specific mechanism of action still remains unclear. Many substances, such as molecular hydrogen and mineral nanoparticles contained in EHW, have been reported to be the action factors of EHWs. In this study, we showed that EHWs exert cytoprotective effects in a hepatocyte culture model by lowering the intracellular levels of ROS and aldehydes, which are toxic substances in ethanol-induced cytotoxicity, through molecular hydrogen. EHW may have a positive effect on the prevention of alcoholic liver disease in the future.

## Data Availability

All data included in this study are available upon reasonable request by contacting the corresponding author.
